# A Novel Class of FKBP12 Ligands Rescues Premature Aging Phenotypes Associated with Myotonic Dystrophy Type 1

**DOI:** 10.3390/cells13231939

**Published:** 2024-11-22

**Authors:** Mikel García-Puga, Gorka Gerenu, Ariadna Bargiela, Jorge Espinosa-Espinosa, Laura Mosqueira-Martín, Maialen Sagartzazu-Aizpurua, Jesús M. Aizpurua, Ainara Vallejo-Illarramendi, Rubén Artero, Adolfo López de Munain, Ander Matheu

**Affiliations:** 1 Cellular Oncology Group, Biogipuzkoa Health Research Institute, Paseo Dr. Beguiristain s/n, 20014 San Sebastian, Spain; mikel.garciapuga@bio-gipuzkoa.eus; 2 Neuroscience Area, Biogipuzkoa Health Research Institute, Biodonostia Institute, 20014 San Sebastian, Spain; gorka.guerenulopetegui@bio-gipuzkoa.eus (G.G.); laura.mosqueiramartin@bio-gipuzkoa.eus (L.M.-M.); ainaravallejo@yahoo.es (A.V.-I.); 3 CIBERNED, CIBER, Carlos III Institute, 28029 Madrid, Spain; 4 Department of Health Sciences, Public University of Navarre (UPNA), Health Sciences Campus, Avda. de Barañain s/n, 31008 Pamplona, Spain; 5 IKERBASQUE, Basque Foundation for Science, 48009 Bilbao, Spain; 6 Translational Genomics Group, University Institute for Biotechnology and Biomedicine (BIOTECMED), University of Valencia, 46100 Burjasot, Spain; ariadna_bargiela@iislafe.es (A.B.); jorge.espinosa@uv.es (J.E.-E.); ruben.artero@uv.es (R.A.); 7 INCLIVA Biomedical Research Institute, 46010 Valencia, Spain; 8 CIBERER, CIBER, Carlos III Institute, 28029 Madrid, Spain; 9 Group of Neuroscience, Departments of Pediatrics and Neuroscience, Faculty of Medicine and Nursing, University of the Basque Country, 20014 San Sebastian, Spain; 10 Joxe Mari Korta R&D Center, Department of Organic Chemistry I, University of the Basque Country, 20014 San Sebastian, Spain; maialen.sagartzazu@ehu.eus (M.S.-A.); jesusmaria.aizpurua@ehu.eus (J.M.A.); 11 Neurology Department, Donostia University Hospital, Osakidetza, 20014 San Sebastian, Spain; 12 Department of Internal Medicine, Faculty of Health Sciences, University of Deusto, 48007 Bilbao, Spain; 13 CIBERFES, CIBER, Carlos III Institute, 28029 Madrid, Spain

**Keywords:** myotonic dystrophy, FKBP12/RyR interaction, oxidative stress, extended longevity, multisystem recovery

## Abstract

**Background:** Myotonic dystrophy type 1 (DM1) is an autosomal dominant disorder clinically characterized by progressive muscular weakness and multisystem degeneration, which correlates with the size of CTG expansion and MBLN decrease. These changes induce a calcium and redox homeostasis imbalance in several models that recapitulate the features of premature tissue aging. In this study, we characterized the impact of a new family of FKBP12 ligands (generically named MPs or MP compounds) designed to stabilize FKBP12 binding to the ryanodine receptors and normalize calcium dysregulation under oxidative stress. **Methods:** Human primary fibroblasts from DM1 patients and control donors, treated with MP compounds or not, were used for functional studies of cell viability, proliferation, and metabolism. The gene expression profile in treated cells was determined using RNA sequencing. The impact of MP compounds in vivo was evaluated in a *Drosophila* model of the disease using locomotor activity and longevity studies. **Results:** The treatment with different MP compounds reversed oxidative stress and impaired cell viability and proliferation, mitochondrial activity, and metabolic defects in DM1-derived primary fibroblasts. RNA sequencing analysis confirmed the restoration of molecular pathways related to calcium and redox homeostasis and additional pathways, including the cell cycle and metabolism. This analysis also revealed the rescue of alternative splicing events in DM1 fibroblasts treated with MP compounds. Importantly, treatment with MP compounds significantly extended the lifespan and improved the locomotor activity of a *Drosophila* model of the DM1 disease, and restored molecular defects characteristic of the disease in vivo. **Conclusions:** Our results revealed that MP compounds rescue multiple premature aging phenotypes described in DM1 models and decipher the benefits of this new family of compounds in the pre-clinical setting of DM1.

## 1. Introduction

Myotonic dystrophy (DM) is the most common inherited form of adult-onset muscular dystrophy. DM is an RNA-dominant disorder caused by the expression of expanded CTG trinucleotide repeats located in the 3′ untranslated region (UTR) of the DM1 protein kinase (*DMPK*) gene in DM type 1 (DM1) [1] and by a tetra-nucleotide CCTG expansion in the zinc finger protein 9 (*CNBP*) gene in DM type 2 (DM2) [2]. DM1 is more common and represents a more severe phenotype than DM2 [3]. In DM1, mutant *DMPK* transcripts containing an expanded CUG tract are retained within the nucleus as discrete foci. Due to their high affinity for CUG expansions, RNA binding proteins, such as the Muscleblind-like (MBNL) family, are sequestered within the CUG-RNA foci, leading to their functional depletion [4]. At the same time, the RNA binding activity of CUG-binding protein 1 (CUG-BP1 or CELF1) rises. These alterations promote alternative splicing changes, impaired gene expression, and additional changes in protein translation, microRNA metabolism, and additional non-coding RNAs, which determine the defects and clinical symptoms observed in the disease [5,6,7]. The length of the triplet expansion increases with age and each generation, and correlates with an earlier age of onset of the disease and a more severe phenotype [8].

Patients with DM1 are characterized by progressive myotonia and muscle weakness and atrophy. Additionally, they display a multisystemic degenerative process characterized by cardiac defects, endocrine disorders, cognitive impairment, insulin resistance, cataracts, increased cancer incidence, neurodegeneration, and premature death [6,9]. Overall, these phenotypes resemble premature aging, leading to the postulate that DM1 is a progeria-like disease [9,10]. Supporting this hypothesis, several pathogenic mechanisms, including senescence accumulation [11,12,13], mitochondrial dysfunction [14], telomere shortening [15], and alteration of autophagy [16], all hallmarks of aging [17], have been associated with DM1 phenotypes.

Despite several ongoing trials, to date there is no treatment to cure or delay the progression of the disease in the clinical setting yet. Supportive treatments, preventative measures, and clinical surveillance are the only options for DM1 patients nowadays [18]. Several novel compounds and strategies have been proposed as anti-DM1 therapy [19,20]. They have primarily been focused on targeting CUG repeats and *DMPK* via reducing its expression and/or preventing interactions with MBNL1. However, it remains to be seen whether any of these strategies are safe and effective in DM1 patients. Indeed, the most advanced therapeutic strategies in the clinical evaluation of DM1 are small molecules that target symptoms or downstream defects, such as metformin, mexiletine, or tideglusib [19,20,21]. Therefore, developing new small molecule compounds targeting critical downstream aspects of the disease remains a priority to complement therapeutics aiming at the root cause of DM1.

Imbalances in calcium and redox homeostasis are common features in aging and neurodegenerative disorders, profoundly contributing to their severity and progression [22,23,24]. Diseases caused by DNA expansion repeats such as DM1 also show defects in calcium and redox homeostasis [24,25,26]. DM1 patients have a chronic increase in cytosolic calcium levels [27,28] and dysregulated calcium metabolism. Interestingly, DM1 patients also display altered alternative splicing events of several genes involved in calcium homeostasis and muscle weakness. In this regard, abnormal skipping in exon 29 of the calcium channel CACNA1S aggravates myopathy in the *HSA^LR^* mouse model of DM1 [29]. In addition, fetal variants of ryanodine receptor type 1 (RyR1), which is involved in the calcium release from intracellular organelles, and SERCA1, which is responsible for calcium transport from the cytosol into the sarcoplasmic reticulum, are significantly increased in skeletal muscles from DM1 patients and in HSA^LR^ mouse [30,31]. Regarding redox homeostasis, DM1 patients present an increase in Reactive Oxygen Species (ROS) levels and oxidative stress biomarkers [32], and an imbalanced oxidant and antioxidant ratio in blood samples [33]. Moreover, in vitro studies showed an increased susceptibility of DM1 cells to oxidative stress, which correlated with CTG repeat expansion [34,35].

Ryanodine receptors (RyRs) are intracellular channels that regulate calcium release from the endoplasmic reticulum into the cytosol of many cell types. They play a role in intracellular calcium homeostasis and are also sensors of the redox state. Consequently, RyRs are essential for numerous cellular functions. RyRs are large complexes comprising several regulatory subunits, including FK506-binding proteins (FKBPs), calmodulin, CaM-dependent kinase II, or protein phosphatases 1 and 2A [36,37]. RyR channels are subject to stress-induced calcium leak, which has been demonstrated to be involved in muscle weakness during extreme fatigue or aging [38], cognitive dysfunction [39], cardiac arrhythmias, and heart failure [40], as well as in muscular dystrophy [41]. Thus, the normalization of RyR channel activity has recently become a therapeutic target for cardiac and muscular disorders [42].

Among the different approaches, compounds to stabilize FKBP12 binding to the RyR, including Rycals, have shown promising results in muscular dystrophies. The better-known agents are benzodiazepines JTV510/K102, S107, and ARM210, which presented cardioprotective effects and improved muscle function and exercise performance in a Duchenne muscular dystrophy mouse model [41,42,43,44,45].

In order to modulate some of the main pathological manifestations induced by DM1, we have designed and synthesized a novel family of 4-arylthioalkyl-1-carboxyalkyl-1,2,3-triazoles (MP compounds) capable of normalizing the FKBP12/RyR1 interaction and modulating calcium dysregulation under oxidative stress conditions [21,46] to further evaluate their impact in experimental models of DM1 disease. Of note, MP compounds showed a similar recovery of the FKBP12/RyR1 interaction compared to S107, with a much higher safety profile [46]. In this work, we studied for the first time the potential impact of novel FKBP12 ligands in DM1, studying the activity of MP compounds in human primary fibroblasts of DM1 patients and a *Drosophila* model of DM1 disease.

## 2. Material and Methods

### 2.1. Compounds

MP compounds have been previously described [46]. In these studies, MP-001 and MP-002 were previously reported as 10b [46] and AHK2 [21], respectively.

### 2.2. Cell Culture

Cultures from different DM1 patients and healthy controls were established and have been previously described [13,14]. Briefly, for the isolation of primary fibroblasts, punch skin biopsies were chopped into 2–3 mm^3^ fragments and placed on a surface moistened with modified Eagle’s medium containing 13% newborn calf serum, 0.4% penicillin/streptomycin (Gibco, Waltham, MA, USA), and 2 mM L-glutamine (Gibco). Flasks were incubated vertically for 3–6 h at 37 °C in a 5% CO_2_ atmosphere and then returned to the horizontal position. Human fibroblasts were cultured in Dulbecco’s Modified Eagle Medium (DMEM, Gibco) containing 10% fetal bovine serum (FBS) (Sigma-Aldrich, St. Louis, MO, USA), 1% L-glutamine (Gibco), and 1% penicillin/streptomycin (Gibco). When indicated, fibroblasts were treated with MP compounds for 72 h. Experiments were performed in early passage cultures (range of 5 to 10 passages). Fibroblasts were tested regularly for mycoplasma contamination. Detailed information of cells used in the manuscript is included in Supplementary Table S1.

### 2.3. Measurement of Cell Growth and Viability and Cell Proliferation

For cell viability, fibroblasts were seeded in 96-well plates followed by treatment with 0.1 µM MP compounds for 72 h. Viable cells were quantified using the modified 3-(4,5-dimethylthiazol-2-yl)-2,5-diphenyltetrazolium bromide (MTT) (M5655, Sigma-Aldrich) assay in six replicates per condition. For P-H3 immunofluorescence assays, 5 × 10^3^ fibroblasts were seeded in immunofluorescence chambers and, after 14–16 h, were treated with MP compounds for 72 h. After that, cells were processed following standard procedures [14]. Primary antibody against phospho Histone H3 Ser10 (ab14955, Abcam, Cambridge, UK) was used.

### 2.4. Total ROS Measurement

A total of 0.1 × 10^4^ fibroblasts was plated in 96-well plates and, after 14–16 h, was treated with 0.1 µM MP compounds for 72 h. Afterwards, ROS-Glo H_2_O_2_ Assay (G8820, Promega, Madison, WI, USA) was performed according to the manufacturer’s instructions to measure H_2_O_2_ concentration. White flat-bottom plates (Corning, Steuben County, NY, USA) were used in a PHERAstar (BMG Labtech, Ortenberg, Germany) luminometer plate reader for the final readout.

### 2.5. Metabolic Measurements

The measurement of OCR and ECAR was performed in XF96 plates with XF Extracellular Flux Analyzer (Agilent, Santa Clara, CA, USA). Fibroblasts were seeded in collagen (BD Biosciences, Franklin Lakes, NJ, USA)-coated XF 96-well plates (Agilent) in octuplicates at 5 × 10^3^ cells/well in 100 μL growth medium and after 14–16 h were treated with 0.1 µM MP compounds for 72 h. Mitochondrial activity was evaluated using the Seahorse XF Cell Mito Stress Test Kit, according to the manufacturer’s instructions (Agilent). Oligomycin (75351, Sigma-Aldrich), FCCP (C2920, Sigma-Aldrich), and Rotenone/Antimycin A, (R8875 and A8674, Sigma-Aldrich) were used at 1.5 μM concentration, after a titration experiment. Cell content was normalized using crystal violet. The post-normalization values of OCR and ECAR reflect the cells’ metabolic activities and the number of cells being measured. Data were further processed using the manufacturer’s calculation matrix.

### 2.6. RNA-Seq Study

RNA was extracted using Trizol (Life Technologies, Carlsbad, CA, USA), and 200 ng of high-quality total RNA was processed for library construction. First, the mRNA was fragmented randomly; then, the cDNA was synthesized. Short fragments were purified and resolved with EB elution buffer for end repair and the addition of single nucleotide A (adenine). After that, the short fragments were ligated with sequencing adapters. Next, the double-stranded cDNA library was completed through size selection and PCR enrichment. During the QC steps, Agilent 2100 Bioanalyzer (Agilent) and ABI StepOnePlus Real-Time PCR System (Applied Biosystems, Waltham, MA, USA) were used to quantify and qualify the sample library. Lastly, the qualified RNA-seq libraries were sequenced using Illumina NovaSeq6000 in CD Genomics (Shirley, NY, USA) after pooling according to its effective concentration and expected data volume. The sequencing was paired-end 150 bp. For the alignment, we used human GRCh38 as the reference genome using the STAR (V2.7.10a) aligner; gene expression was quantified using RSEM (V1.3.1) and differential expression for each gene was performed using the edgeR package (v 4.0.3). Genes with log2 fold change over or under 1 and an adjusted *p*-value under 0.05 were considered significantly altered. Splicing analysis was performed using Vast-Tools (V2.3.0). We pooled the reads for each state using the merge option and considered a change in Percentage of splice In (PSI) significant if it was above 15 percent and the adjusted *p*-value was under 0.05 using the compare module.

### 2.7. mRNA Expression Analysis

Total RNA was extracted using TRIzol (Life Technologies). Reverse transcription was performed using random priming and the Maxima First Strand cDNA Synthesis Kit for RT-qPCR with dsDNase (Thermo Fisher Scientific, Waltham, MA, USA), according to the manufacturer’s guidelines. Quantitative PCR was performed in a CFX384 thermocycler (Bio-Rad, Hercules, CA, USA) using Power SYBR Green PCR Master Mix (Thermo Fisher Scientific), 10 mM of each primer, and 20 ng of cDNA. Variations in RNA input were corrected by analyzing the expression of *GAPDH* (human) or rp49 (*Drosophila*) as housekeeping genes. The ΔΔC_T_ method was used for relative quantification. Primer sequences are in Supplementary Table S5.

### 2.8. Transgenic Drosophila Melanogaster

We used the transgenic Mhc-Gal4 UAS-i(CTG)480 flies [47], referred to as REC2. Flies were housed at 70% humidity and 12 h/12 h light/dark cycles. All crosses were carried out at these conditions with standard fly food or plus MP-002 10 or 100 µM for the treated group. For the longevity study, 50–80 newly emerged flies were collected in freshly prepared tubes containing standard feed with or without MP-002. Males and females were kept in separate tubes at 25 °C. The number of dead flies was scored daily. Flies were transferred to new tubes twice a week. Survival curves were obtained using the Kaplan–Meier method, and the results were analyzed using the log-rank test. For locomotor activity, 20- and 25-day old flies were analyzed in groups of five. They were placed in a tube with an 8 cm mark from the bottom. The assays were recorded and archived. The number of flies that passed the 8 cm mark in 10 s was counted (3 times/tube). mRNA was isolated from the thorax of a mix of 6 flies per sample and condition.

### 2.9. Statistics

Data are presented as mean values ± S.E.M., with the number of experiments (n) in parentheses. Unless otherwise indicated, statistical significance (*p*-values) was calculated using the Student’s *t*-test. Asterisks (*^≠^*, *, **, and ***) indicate statistical significance (*p* < 0.1, *p* < 0.05, *p* < 0.01, and *p* < 0.001, respectively).

## 3. Results

### 3.1. MP Compounds Restore Cell Growth and ROS Levels in DM1 Primary Fibroblasts

**Figure 1:**
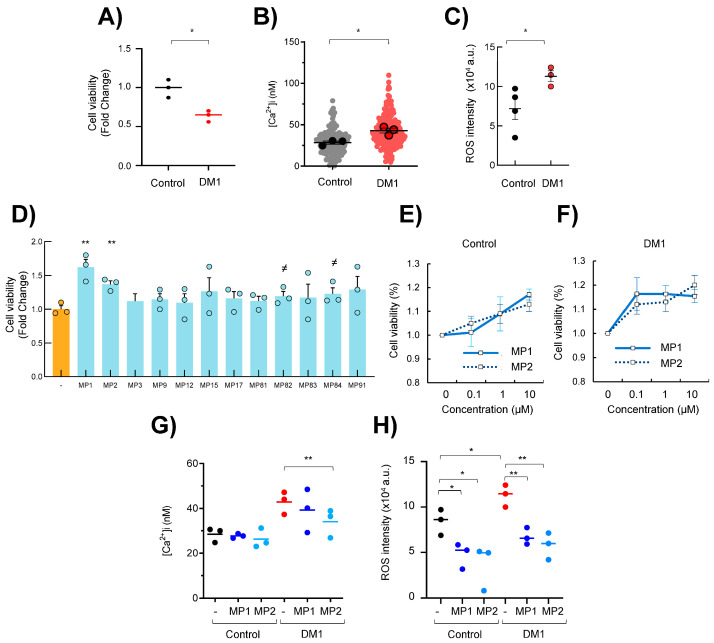
MP compounds restore DM1 cell viability and redox homeostasis. (**A**–**C**) Cell viability, intracellular calcium measurement, and ROS levels in fibroblasts derived from DM1 patients and controls. Values in DM1 are relative to controls. Dots represent mean values from control and patient individuals. Delineated dots in B represent average values from at least 30 cells, represented as non-delineated dots. (**D**) Cell viability of DM1 fibroblasts after treatment with 0.1 µM of indicated MP compounds for 72 h (n = 3, different individuals). (**E**,**F**) Cell viability of control and DM1 fibroblasts after treatment with 0.1, 1, and 10 µM of MP-001 and MP-002 for 72 h (n = 3). (**G**) Intracellular calcium measurement in same conditions as ((**E**); n = 3). (**H**) ROS levels in fibroblasts derived from DM1 patients and controls after treatment with 0.1 µM of MP-001 and MP-002 for 72 h (n = 3). *^≠^ p* < 0.1, * *p* < 0.05, and ** *p* < 0.01.

First, we examined cell growth and viability in primary fibroblasts from DM1 patients and healthy controls of similar age performing MTT studies. The comparison between both groups revealed a significant reduction of ~50% in DM1 fibroblasts (Figure 1A). The impaired cell growth was paralleled by a ~50% increase in intracellular calcium levels (Figure 1B) and a marked accumulation in ROS levels in DM1 fibroblasts compared to healthy controls (Figure 1C). In this context, we tested the effect of MP compounds on cell growth and viability. For this, we cultured DM1 fibroblasts with a single dose of 0.1 µM of 12 MP compounds for 72 h. Interestingly, several MP agents improved the impaired cell viability of DM1 fibroblasts (Figure 1D). Among them, MP-001 and MP-002 were the compounds that restored cell growth and viability more robustly. Therefore, we treated cells with increasing concentrations of 0.1, 1, and 10 µM of MP-001 and MP-002 for 72 h. These compounds did not exhibit cell toxicity and increased the cell viability in DM1 fibroblasts from the minimal dose of 0.1 µM and 10 µM in controls (Figure 1E,F).

Next, we measured the calcium levels and found that the treatment with MP-002 significantly rescued the increased levels in DM1 cells (MP-001 trended towards normalization but did not reach statistical significance), whereas no effect was detected in control cells (Figure 1G). Regarding ROS levels, the treatment with the minimal dose of 0.1 µM of MP-001 and MP-002 notably decreased them in both genotypes, but especially in DM1, reaching intensity values like those of the controls (Figure 1H). These results reveal that MP compounds rescued cell growth in DM1, paralleling the normalization of ROS levels.

### 3.2. MP Compounds Normalize Premature Aging Phenotypes in DM1 Primary Fibroblasts

**Figure 2:**
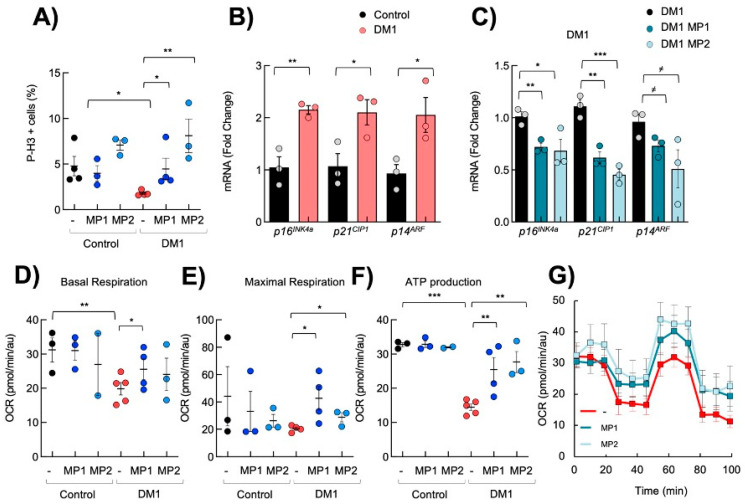
MP compounds restore DM1 cell proliferation and metabolism. (**A**) Quantification of the number of P-H3-positive cells in independent control and DM1 fibroblasts (n > 3) after treatment with 0.1 µM of MP-001 and MP-002 for 72 h. (**B**) mRNA levels of *p16^INK4A^*, *p21^CIP1^,* and *p14^ARF^* in control and DM1 fibroblasts (n ≥ 3), (**C**) and after treatment with 0.1 µM of MPs for 72 h. (**D**–**F**) Quantification of basal, maximal respiration, and ATP production in controls and DM1 (n > 3) fibroblasts after 0.1 µM MP compound treatment. (**G**) Kinetic normalized OCR response in DM1 fibroblasts in the absence or presence of 0.1 µM of MP-001 and MP-002. *^≠^ p* < 0.1, * *p* < 0.05, ** *p* < 0.01, and *** *p* < 0.001.

DM1 fibroblasts display decreased proliferative capacity, mitochondrial function and metabolism, and enhanced senescence [14], all hallmarks of cellular aging [48]. Next, we investigated the functional impact of MP-001 and MP-002 on those processes. First, as previously described, we found fewer phospho-Histone H3 (pH3)-positive cells in DM1 cells than in controls (Figure 2A). Notably, the treatment with 0.1 µM of MP-001 and MP-002 increased the number of pH3 positive cells by almost 3- and 4-fold in DM1 cells, respectively, reaching the levels of control cells (Figure 2A). Impaired proliferation and enhanced senescence in DM1 cells have been linked to elevated levels of p16^INK4A^ and p21^CIP^ cell cycle inhibitors [10,49]. Consistent with this, we also found an increased expression of p16^INK4A^, p21^CIP^, and *p14^ARF^*, an additional cell cycle inhibitor closely related to p16^INK4A^ in DM1 cells (Figure 2B). Interestingly, MPs reduced the expression of *p16^INK4A^*, *p14^ARF^*, and *p21^CIP^* in DM1 fibroblasts (Figure 2C).

Next, we evaluated cellular metabolism and mitochondrial activity using SeaHorse. First, we confirmed that DM1 fibroblasts displayed a reduced basal oxygen consumption rate, maximal respiration, and ATP production (Figure 2D–F). Interestingly, 0.1 µM of MP-001 and MP-002 improved the basal oxygen consumption rate and maximal respiration of DM1 fibroblasts (Figure 2D,E). Moreover, MP-001 and MP-002 also significantly restored ATP production via oxidative phosphorylation with no, or a discrete, effect in control fibroblasts (Figure 2F,G). In most experiments, the effect of MP-002 was stronger than other MP product variants, thus becoming the most promising candidate within this family.

### 3.3. Transcriptomic Analysis Reveals That MP-002 Normalizes Altered Molecular Pathways in DM1 Primary Fibroblasts

**Figure 3:**
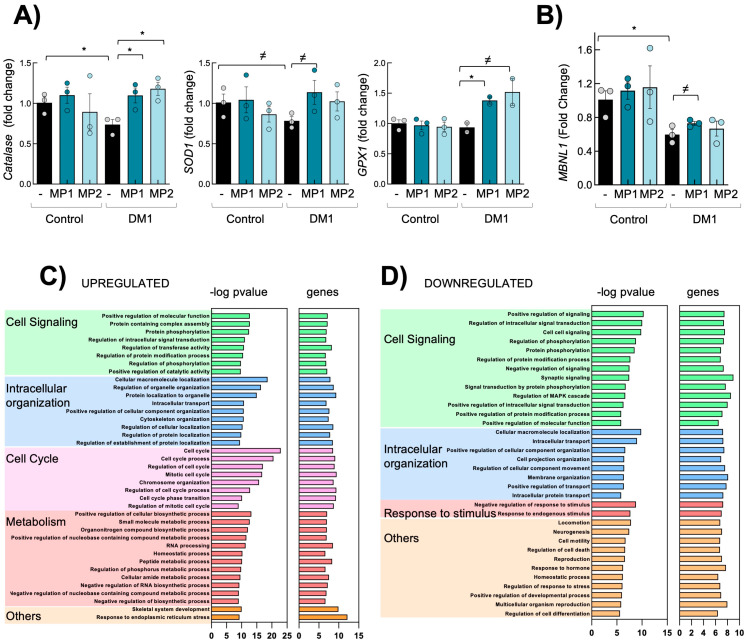
MP-002 rescues molecular alterations in DM1 fibroblasts. (**A**,**B**) mRNA levels of indicated genes in control and DM1 fibroblasts (n = 3). (**C**,**D**) Bar plot of the -log10 (p-value) of the significantly upregulated and downregulated GO terms in DM1 fibroblasts treated with MP-002 (n = 3). *^≠^ p* < 0.1, and * *p* < 0.05.

To comprehensively understand the impact of MP compounds in DM1 fibroblasts, we performed an unbiased high-throughput RNA-sequencing (RNA-seq) in three independent DM1 fibroblasts in response to MP-002 for 72 h. The transcriptome-wide study showed many genes altered in DM1 cells cultured with MP-002. Among them, genes related to the RyR channel and calcium homeostasis, such as Calmodulin, CAMK2N1 (calcium/calmodulin-dependent protein kinase II inhibitor 1), and CAMK2D (calcium/calmodulin-dependent protein kinase II delta) [24,36] were elevated (Supplementary Tables S2 and S3). In this line, genes related to redox homeostasis, such as Sestrins (SESN1, SESN2) or Superoxide dismutase 2 (SOD2), were altered, further validating the activity of MP compounds on the normalization of calcium and redox homeostasis. In this line, q-RTPCR validations confirmed the restoration of genes related to calcium and redox homeostasis such as *SOD1*, *Catalase*, or *GPX1* genes (Figure 3A). MBNL depletion is a primary contributor to DM1 phenotypes. In this line, MPs also partially rescued the expression of *MBNL1* (Figure 3B). Moreover, Gene Ontology (GO) enrichment analysis showed that cell cycling, metabolism, and cell signaling were the processes most significantly upregulated in MP-002-treated fibroblasts compared to non-treated cells (Figure 3C and Supplementary Tables S2 and S3). On the contrary, cell-signaling genes associated with response to stimulus and intracellular organization were downregulated with the treatment of MP-002 (Figure 3D and Supplementary Tables S2 and S3).

### 3.4. Transcriptomic Analysis Reveals That MP-002 Normalizes Altered Molecular Pathways and Splicing Defects in DM1 Primary Fibroblasts

**Figure 4:**
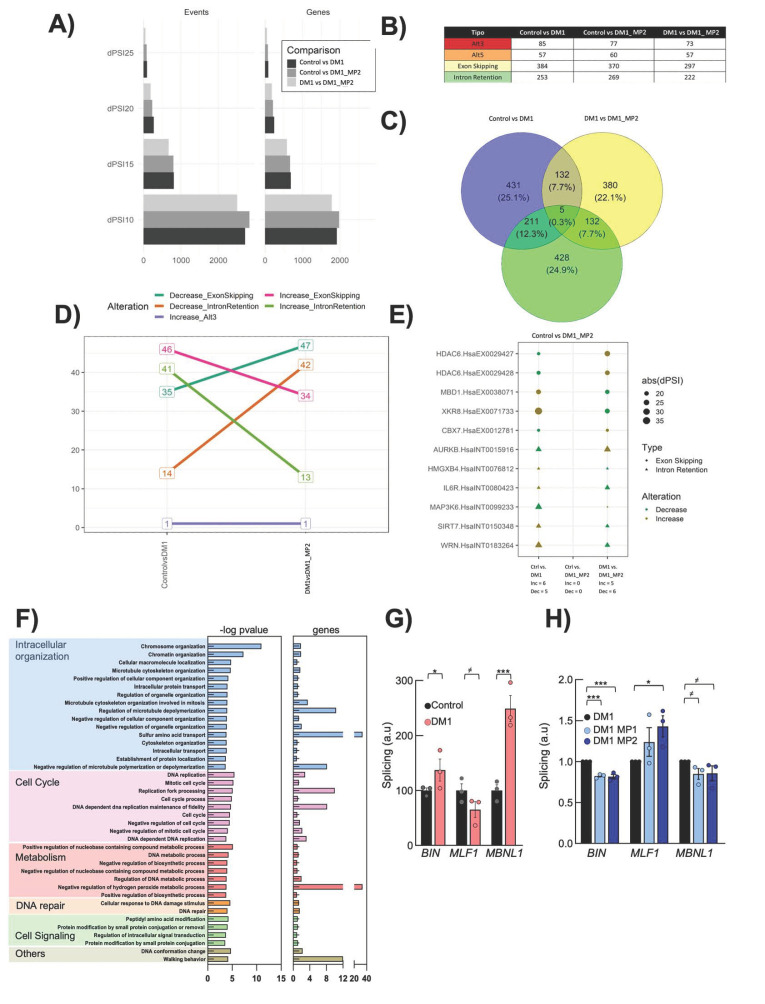
MP-002 rescues splicing events. (**A**) Quantity of splicing events in each dPSI. (**B**) Quantity of types of splicing events in each comparison. (**C**) Venn diagram of common genes in each comparison. (**D**) Change in direction of the splicing events affected by the treatment. (**E**) Point plot of the top 10 recovered genes. (**F**) Bar plot of the -log10 (p-value) of the significantly altered GO terms from genes with aberrant splicing events in DM1 fibroblasts treated with MP-002 (n = 3). (**G**,**H**) Quantification of splicing in *BIN*, *MLF1*, and *MBNL1* in control and DM fibroblasts and restoration after MP treatment (n = 3). *^≠^ p* < 0.1, * *p* < 0.05, and *** *p* < 0.001.

The abnormal regulation of alternative splicing is a molecular hallmark of DM1 [50]. Therefore, we characterized the impact of MPs in alternative splicing defects. We performed a splicing event analysis over RNA-seq data obtained from three biological replicas of the Control, DM1, and DM1 treated with MP-002 samples. We performed three comparisons: Control vs. DM1, to identify disease related splicing events; DM1 vs. DM1 MP, to identify the effect of our treatment in the cell model of the disease; and Control vs. DM1 MP, to assess the differences after treatment. We tested different cut points with 10, 15, 20, and 25 dPSI for each comparison, from which we selected dPSI15 since it presented enough splicing events (779) without identifying a lot of smaller events (2585) that could possibly mask biologically relevant information (Figure 4A). The conformation of the splicing events in all comparisons is presented in the Figure 4B. Notably, we identified 132 events altered by the disease and by the effects of MP treatment and 5 events altered by all three comparisons; these 137 splicing events were considered the specific effect of the treatment over the disease model (Figure 4C). To further understand the alteration of these events, we analyzed the direction of the alterations for each comparison, finding that 132 events presented a contrary direction of the one present in the Control vs. DM1 suggesting a recovery (Figure 4D). Since none of the events was present in the comparison of Control vs. DM1 MP we could indicate a full recovery corresponding to 16.9% of all the altered events in the Control vs. DM1 comparison. The remaining five events corresponded to non-specific effects and covered 0.6% of the altered splicing events present in the Control vs. DM1 comparison. All 137 events are presented in Supplementary Table S4, and among them, we present the top 10 genes and 11 events recovered by MP treatment (Figure 4E). Notably, GO enrichment analysis of the rescued alterations showed processes such as cell cycling, metabolism, intracellular organization, cell signaling, and DNA repair (Figure 4F), revealing a correlation between the improvements in gene expression and splicing alterations. Moreover, MP-002 rescued alternative splicing events in several genes previously linked to the pathobiology of the disease, such as *MLF1*, *CAPN3*, *ANK2*, *BIN1*, *ARFGAP2*, and *FHOD1* [51]. Of note, experimental validation confirmed the restoration of *MBNL1*, *MLF1*, and *BIN1* genes with MP treatment (Figure 4G,H). Moreover, among the restored genes, there were several which have been related to aging (Supplementary Figure S1).

### 3.5. MP-002 Rescues Critical Phenotypes in a Drosophila Model of DM1 Disease

Next, we extended the results of MP compounds in vivo and characterized the effect of MPs in a *Drosophila melanogaster* model of DM1 (REC2), which expresses 480 interrupted CUG repeats in somatic muscle under the *Mhc-Gal4* driver [34,37,47]. In this case, we selected MP-002, as we had previously shown that this compound had the most potent effect in vitro and crossed the blood–brain barrier.

**Figure 5:**
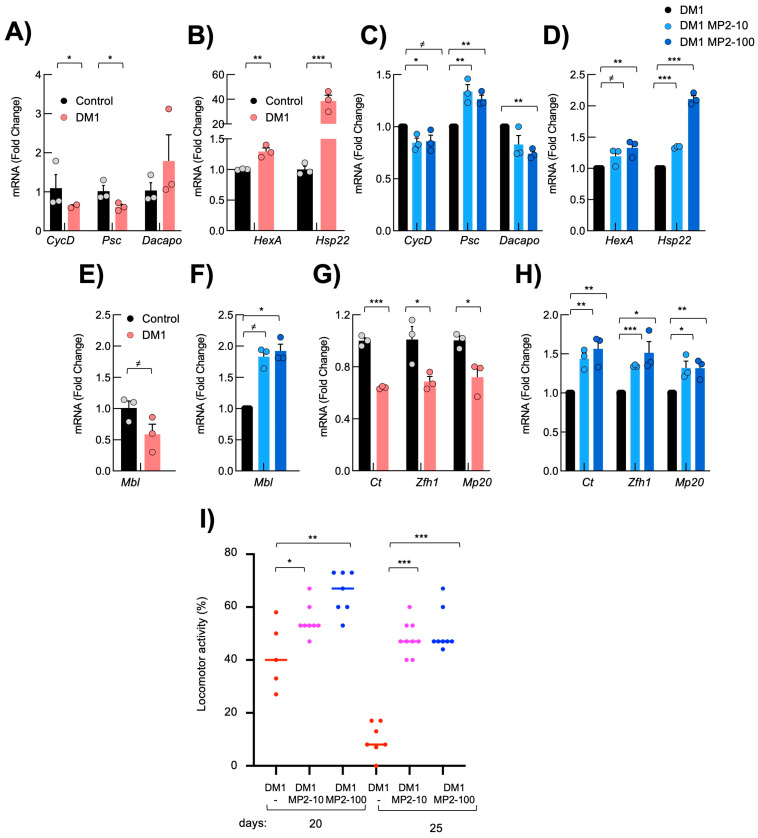
MP-002 restores molecular and functional defects in DM1 *Drosophila melanogaster*. (**A**–**D**) mRNA levels of indicated genes in the thorax of DM1 and control fruit flies (n = 3, each point represents a pool of six flies). (**E**–**H**) mRNA levels of indicated genes from the thorax of DM1 fruit flies in the presence of 10 and 100 µM of MP (n = 3, each datapoint comes from a pool of six flies). (**I**) Locomotor activity of non-treated DM1 (n = 50) flies or in the presence of 10 and 100 µM of MP (n = 50) at the indicated time points. Student *t*-test values are at 20 days *p* = 0.02 and *p* = 0.002, and at 25 days *p* <0.0001 compared to non-treated flies. *^≠^ p* < 0.1, * *p* < 0.05, ** *p* < 0.01, and *** *p* < 0.001.

First, we measured the expression of several genes involved in cell proliferation and mitochondrial metabolism in the thoraces of adult DM1 and control flies, mainly including indirect flight muscles. This comparison showed that *Dacapo* (homolog of p21^CIP^/p27^KIP^) was elevated, whereas *Psc* (homolog of BMI1, upstream negative regulator of p21^CIP^) and *CyclinD* were reduced in DM1 flies (Figure 5A). Regarding metabolism, *HexokinaseA* and *Hsp22* were elevated in DM1 flies (Figure 5B), likely as a compensatory mechanism consequence of the deleterious accumulation of stress and damage [52]. Notably, MP-002 decreased the levels of *Dacapo,* whereas it increased *Psc* and *CyclinD* (Figure 5C). At the same time, the levels of *HexokinaseA* and *Hsp22* were further increased (Figure 5D).

Reduced *Mbl* levels have been previously described in the *REC2* DM1 *Drosophila* model [16]. Consistent with this, *Mbl* levels were reduced in DM1 flies, while MP-002 increased *Mbl* expression, reaching the levels of the controls (Figure 5E,F). Since muscle weakness is a major characteristic of the disease, we tested the impact of MP compounds on muscle function markers. First, we measured the expression of *Ct*, *Zflh1*, and *Mp20*, all genes related to muscle activity. The reduced expression of the three genes in the muscle of thoraces of DM1 flies was significantly rescued with MP-002 (Figure 5G,H). The restoration of their levels upon MP-002 administration was dose-dependent, except for Mp20, suggesting a direct effect of MP compounds in muscle tissue.

To test whether the increased expression of muscle genes was correlated with improved functional locomotion, we studied the motility and flight capacity in DM1 flies upon administering MP compounds. Notably, MP-002 significantly increased the motility of DM1 flies. The percentage of flies with climbing ability at 25 days was 10% in non-treated DM1 flies compared to 40 and 50% with 10 and 100 µM of MP-002 (Figure 5I). In summary, these results confirm that MP-002 restored muscle function in an in vivo model of the disease, and these rescues were correlated with increased *Mbl* levels.

### 3.6. MP Compounds Rescue Reduced Longevity in a Drosophila Model of DM1 Disease

**Figure 6:**
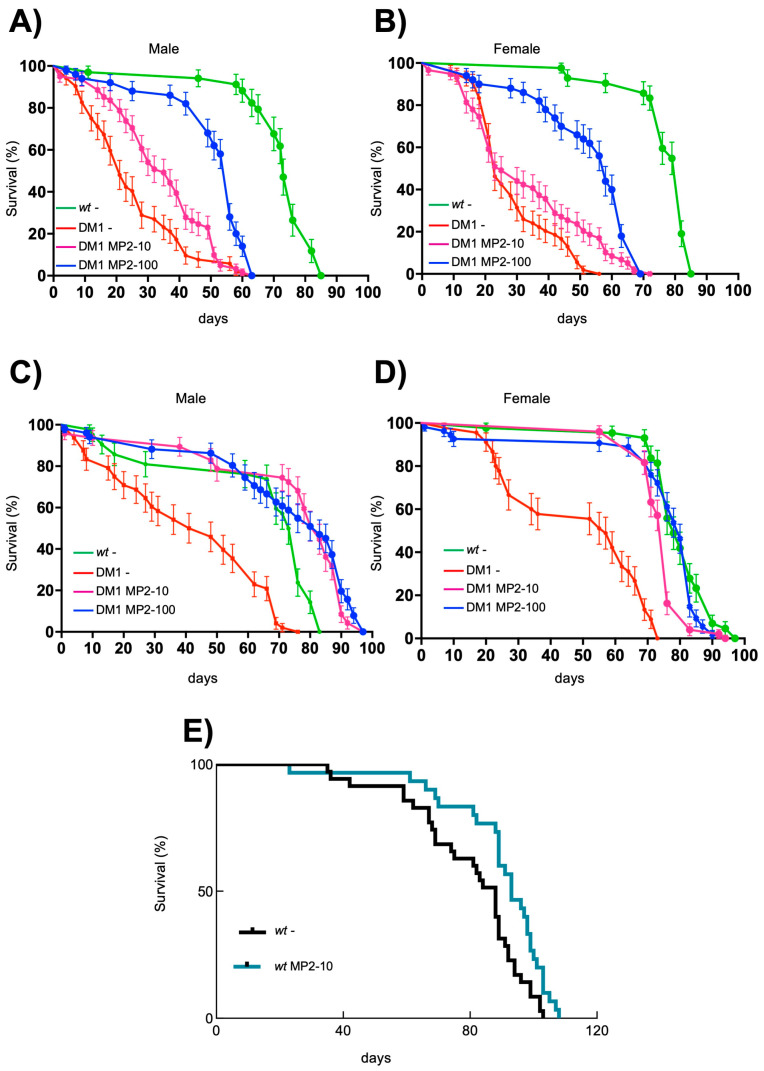
MP-002 extends lifespan in DM1 *Drosophila melanogaster*. (**A**,**B**) Survival curves of control (*wt*), non-treated DM1 (DM1 -), and DM1 flies in the presence of 10 µM (DM1 MP2 10) and 100 µM (DM1 MP2 100) of MP-002 treated since larval stage divided by sexes; male (**A**), female (**B**), (n = 100). LogRank values are *p* < 0.0001 for both sexes compared to non-treated flies. (**C**,**D**) Survival curves of control flies, DM1 flies non-treated, and DM1 flies in the presence of 10 and 100 µM of MP-002 treated since adulthood divided by sexes; male (**C**), female (**D**), (n = 100). LogRank values are *p* < 0.0001 for both sexes. (**E**) Survival curve of control flies, non-treated, or in the presence of 10 µM of MP-002 treated since adulthood (n = 50). LogRank value is *p* < 0.05.

DM1 patients have reduced life expectancy due to multisystem defects, including muscle wasting and cardiac deficiencies [53], the main known targets of Rycal compounds [42]. Similarly, the *REC2* DM1 *Drosophila* model also displays a reduced lifespan [16]. Consistent with this, our studies showed an over 50% reduction in the median and maximal lifespan in both sexes in *REC2* DM1 compared to *wt* flies (Figure 6A–D). In this context, we performed a longevity curve experiment, treating DM1 flies with two doses of 10 and 100 µM throughout larval development and adulthood. Interestingly, MP-002 administration since the larval stage significantly extended the lifespan of DM1 flies in a dose-dependent manner in both sexes (Figure 6A,B). Moreover, we investigated the effect of MP-002 in adult flies, i.e., flies that emerged from untreated DM1 larvae, to assess whether the phenotypes were reversed once already manifested in the animal. In this case, MP-002 extended by more than double the lifespan of DM1 flies, reaching the survival levels of control flies in both sexes. Thus, MP-002 extended the median survival from 40 to more than 80 days in males and from 45 to more than 70 days in female flies (Figure 6C,D). Since MPs have positively affected some activities of control fibroblasts, we studied whether they could influence longevity in *wt* flies. MP-002 extended both the median and maximal longevity in *wt* adult flies, suggesting that MP compounds could exert beneficial effects on key aging-related pathways (Figure 6E). In summary, we revealed the benefit of MP-002 in critical DM1 phenotypes in vivo.

## 4. Discussion

MP compounds are a novel family of 4-arylthiometyl-1,2,3-triazoles designed to normalize calcium dysregulation under nitro-oxidative stress. Confirming previous experiments [46], we observed a restoration of calcium and redox homeostasis in DM1 cells in vitro and in vivo. A limitation of our study is that the detailed and precise molecular mechanism was not determined. This limitation, however, is characteristic of all the compounds directed against ryanodine receptors [54]. In this regard, the normalization of calcium and redox homeostasis has been shown to regulate cell metabolism and proliferation, and has been linked to muscle activity and longevity [55,56]. Several observations suggest that the results obtained are better explained by the rescue of alterations in redox homeostasis, namely, (i) the reduction in ROS levels was higher than calcium in DM1 fibroblasts; (ii) MP compounds exerted some protective effects at the cellular and physiological level in *wt* cells, which correlated with decreased ROS accumulation; and (iii) the free radical hypothesis of oxygen toxicity, which is supported by multiple studies in different animal models, proposes that aging is the consequence of the harmful activities of free radicals endogenously formed during normal metabolic processes. In line with this, normal aging is associated with the oxidation of a wide range of cellular proteins, and it has been proposed that ROS selectively modify proteins, ultimately resulting in a loss of calcium homeostasis. Over time, oxidative damage to cells and tissues accumulates, impairing cellular function and leading to aging. ROS-induced damage is believed to contribute to a variety of age-related conditions such as neurodegeneration, cardiovascular disease, diabetes, and cancer. Mitochondria are primary sources of ROS production, and we have previously reported that mitochondrial metabolism is altered in DM1 fibroblasts [14]. The accumulation of toxic RNA in DM1 cells can damage mitochondria and interfere with their normal function, leading to mitochondrial dysfunction and increased ROS generation. Mitochondrial damage exacerbates oxidative stress, creating a vicious cycle of further cellular injury. [55,56].

It is known that most molecular DM1 alterations stem from the depletion of MBNL proteins and the limited availability of MBNLs is a primary contributor to DM1 phenotypes. Indeed, the loss of MBNL proteins is responsible for myotonia in the context of human skeletal muscle and loss of *MBNL1* function accounts for more than 80% of mis-splicing events and nearly 70% of expression defects in a murine model expressing 250 CTG repeats. In this regard, MP-002 rescued *Mbl* expression. The rescue of normal splicing patterns is a promising therapeutic approach in DM1 such as through antisense oligonucleotides (ASOs) or small molecule modulators. It is important to emphasize that MP compounds restored different types of mis-splicing events affecting genes and processes previously described to be involved in the pathobiology of the disease including *MBNL1* [51]. The ability to restore splicing events differs from rapamycin [46], a potent anti-aging compound [57], with which MP compounds share some characteristics. Rapamycin is also a ligand of FKBP12; however, it displaces FKBP12 from RyR1 channels, resulting in increased calcium leakage through RyR1 from the sarcoplasmic reticulum into the cytosol [58]. It also exerts several functions, including mTOR inhibition and autophagy modulation, which might explain the different activities in DM1, including negative effects in cell proliferation [59] and an improvement in muscle strength [43].

With age, the capacity for cellular proliferation tends to decline, contributing to the overall aging process and the development of age-related diseases. This decline is not limited to a reduction in the number of dividing cells, but also includes dysregulated cell cycle control, altered cellular responses to stress, and the accumulation of DNA damage. The interplay between cellular proliferation and aging is complex and involves a variety of molecular pathways, many of which are tightly regulated to balance cell survival, function, and tissue homeostasis. Here, we described a reduction in cell proliferation in DM1 fibroblasts and alterations in genes related to proliferation, supporting the idea of a premature aging phenotype in DM1. This reduction in the proliferative capacity was rescued with MP compounds.

In summary, our results revealed that the MP compounds, a new family of FKBP12 ligands, rescued multiple and critical DM1 phenotypes in two different models of the disease: elevated ROS production, impaired proliferation and metabolism in human primary fibroblasts in vitro; and reduced longevity and impaired locomotor activity in a *Drosophila* model in vivo. Notably, all these phenotypes are critical in aging. The results of RNA-seq analysis and the additional molecular validations indicated that MPs regulate the original targets and influence additional molecular and signaling pathways important for the aging process.

## Data Availability

The data discussed in this publication have been deposited in NCBI’s Gene Expression Omnibus and are accessible in GSE186494.

## Supplementary Materials

The following supporting information can be downloaded at: https://www.mdpi.com/article/10.3390/cells13231939/s1, Table S1: Characteristics of individuals from whom fibroblasts have been used; Table S2: List of downregulated genes; Table S3: List of uregregulated genes; Table S4: Dysregulated splicing events; Table S5: List of primers used in the manuscript; Figure S1: AHK rescue splicing events in aging related genes.

## Abbreviations

Myotonic dystrophy type 1 (DM1), MP compounds (MPs), DM1 protein kinase (*DMPK*), muscleblind like splicing regulator 1 (MBNL), CUG-binding protein 1 (CUG-BP1 or CELF1), ryanodine receptor type 1 (RyR1), Reactive Oxygen Species (ROS).
