# Supplementary material for: A Novel Class of FKBP12 Ligands Rescues Premature Aging Phenotypes Associated with Myotonic Dystrophy Type 1
Source: Cells. 2024 Nov 22;13(23):1939. doi: 10.3390/cells13231939 (PMC11639790; doi:10.3390/cells13231939)
Supplement: Supplementary file 1 [file cells-13-01939-s001.zip › Garc¿¬a-Puga et al., figures Suppl.pptx]

## Slide 1
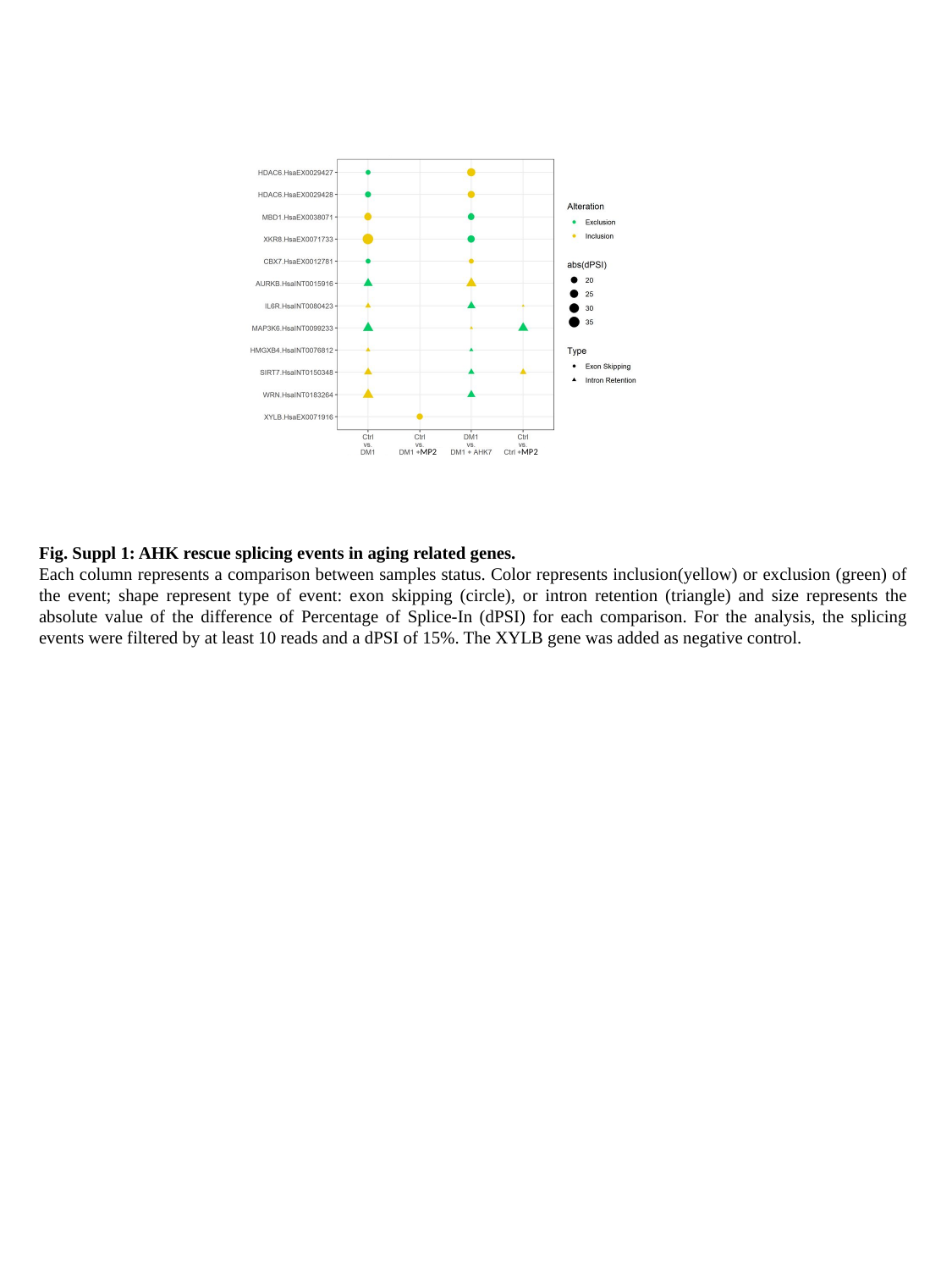

MP2
MP2
Fig. Suppl 1: AHK rescue splicing events in aging related genes.
Each column represents a comparison between samples status. Color represents inclusion(yellow) or exclusion (green) of the event; shape represent type of event: exon skipping (circle), or intron retention (triangle) and size represents the absolute value of the difference of Percentage of Splice-In (dPSI) for each comparison. For the analysis, the splicing events were filtered by at least 10 reads and a dPSI of 15%. The XYLB gene was added as negative control.
