# Supplementary material for: A Novel Class of FKBP12 Ligands Rescues Premature Aging Phenotypes Associated with Myotonic Dystrophy Type 1
Source: Cells. 2024 Nov 22;13(23):1939. doi: 10.3390/cells13231939 (PMC11639790; doi:10.3390/cells13231939)
Supplement: Supplementary file 1 [file cells-13-01939-s001.zip › Supp Table 1 Garcia Puga et al.pdf]

**Table 1. Characteristics of individuals from whom fibroblasts have been used**

| Fibroblast Cell Line | Status | Gender | MIRS | CTG (n) in fibroblast (early passage) | Age at biopsy (years) |
|---|---|---|---|---|---|
| C1 | Control | M | | | 49 |
| C2 | Control | F | | | 48 |
| C3 | Control | M | | | 27 |
| C4 | Control | M | | | 29 |
| C5 | Control | M | | | 46 |
| C6 | Control | ? | | | 52 |
| DM1-1 | DM1 | M | 3 | 221 | 71 |
| DM1-2 | DM1 | M | 5 | 714 | 50 |
| DM1-3 | DM1 | M | 3 | 671 | 56 |
| DM1-4 | DM1 | F | 2 | 243 | 45 |
| DM1-5 | DM1 | F | 2 | 648 | 44 |
| DM1-6 | DM1 | M | 3 | 588 | 56 |
| DM1-7 | DM1 | F | 4 | 750 | 34 |

MIRS (Muscle impairment rating scale)
